# Supplementary material for: Electrohydraulic musculoskeletal robotic leg for agile, adaptive, yet energy-efficient locomotion
Source: Nat Commun. 2024 Sep 9;15:7634. doi: 10.1038/s41467-024-51568-3 (PMC11385520; doi:10.1038/s41467-024-51568-3)
Supplement: Supplementary file 2 — Supplementary Information [file 41467_2024_51568_MOESM2_ESM.pdf]

# Supplementary Information to

# "Electrohydraulic musculoskeletal robotic leg for agile, adaptive, yet energy-efficient locomotion"

Thomas J. K. Buchner[1,†], Toshihiko Fukushima[2,†], Amirhossein Kazemipour[1], Stephan-Daniel Gravert[1], Manon Prairie[1], Pascal Romanescu[1], Philip Arm[1,3], Yu Zhang[1,2], Xingrui Wang[2], Steven L. Zhang[2], Johannes Walter[2], Christoph Keplinger[2,4,5,*], Robert K. Katzschmann[1,6,*]

[1]Soft Robotics Lab, D-MAVT, ETH Zurich, 8092 Zurich, Switzerland
[2]Robotic Materials Department, Max Planck Institute for Intelligent Systems; 70569, Stuttgart, Germany
[3]Robotic Systems Lab, D-MAVT, ETH Zurich, 8092 Zurich, Switzerland.
[4]Paul M. Rady Department of Mechanical Engineering, University of Colorado Boulder; Boulder, CO 80309, USA
[5]Materials Science and Engineering Program, University of Colorado Boulder; Boulder, CO 80309, USA
[6]ETH AI Center, ETH Zurich, 8050 Zurich, Switzerland

[†]These authors contributed equally to this work.

*Corresponding author(s):
rkk@ethz.ch; ck@is.mpg.de

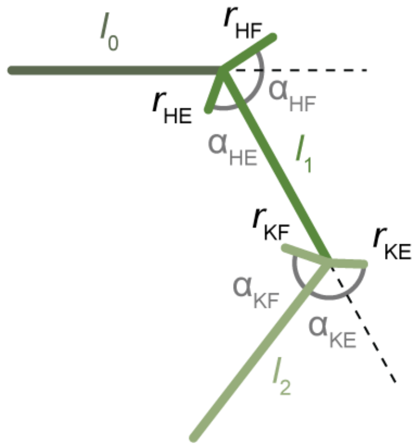


| Parameter | Value |
|---|---|
| $l_0$ | 271 mm |
| $l_1$ | 283 mm |
| $l_2$ | 187 mm |
| $r_{HF}$ | 20 mm |
| $r_{HE}$ | 20 mm |
| $r_{KF}$ | 20 mm |
| $r_{KE}$ | 20 mm |

| Parameter | Value |
|---|---|
| $\alpha_{HF}$ | 143.0° |
| $\alpha_{HE}$ | 38.4° |
| $\alpha_{KF}$ | 38.4° |
| $\alpha_{KE}$ | 116.0° |

**Supplementary Figure 1 | Leg geometry.** The leg's basic geometry with measurements of the lever arms and angles is required to simulate its kinematics. The actuators are fixed to one end of the carbon fiber rods using a 3D-printed attachment. The distance to the bone's central axis is at least 12 mm. Actuators are stacked atop each other, increasing that distance. A tendon made from a fishing line connects the other end to the lever arm.

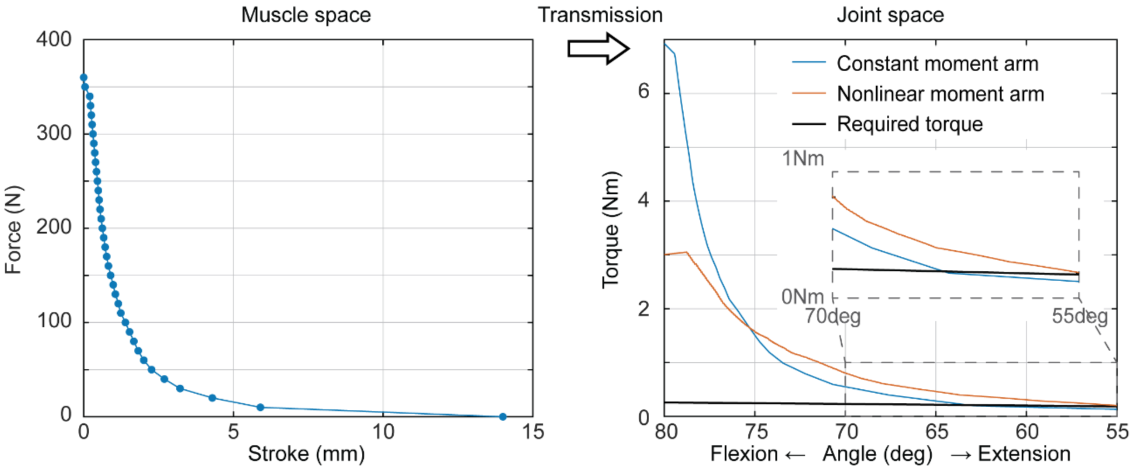


**Supplementary Figure 2 | Transmission using constant or nonlinear moment arm.** Left: Force-strain profile of the Peano-HASEL artificial muscle for the knee extension. Right: The torque-angle profile is calculated from the muscle profile and transmission geometry after being transmitted by either a constant or a nonlinear moment arm. The black line represents quasi-static torque at each knee angle to maintain the desired posture during squatting.

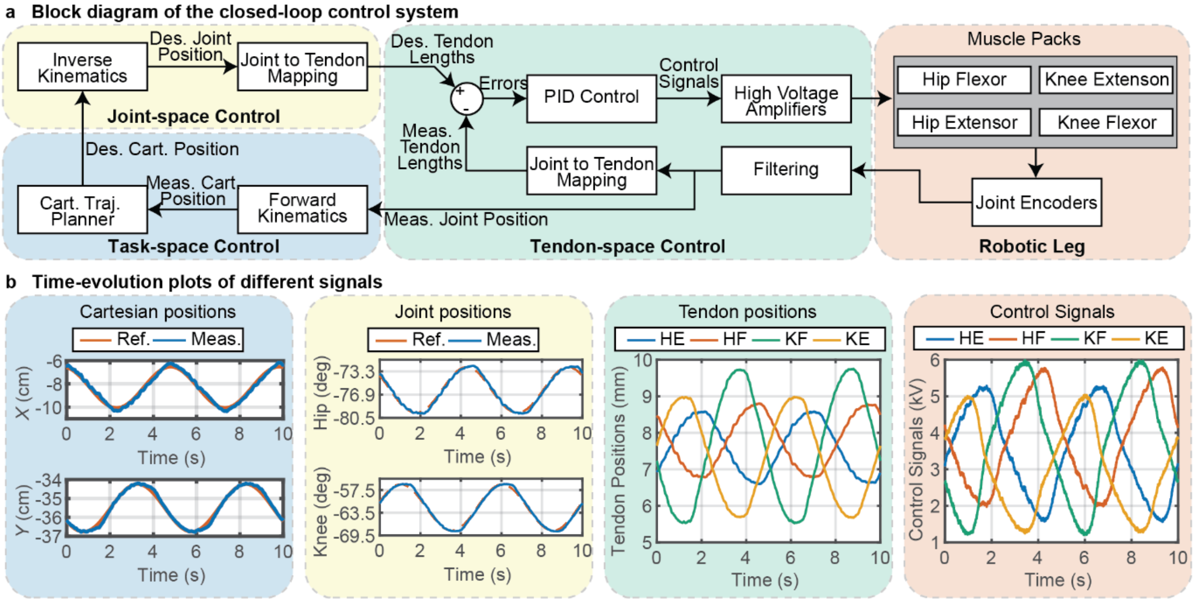


**Supplementary Figure 3 | Description of the closed-loop control system. a,** The control block diagram illustrates the interconnected components of high-level and low-level control. **b,** Trajectory tracking of the leg's tip in task space following an ellipse trajectory with a period of 5 s. The signal transformations from Cartesian space to joint space and then to tendon space are highlighted. The tendon positions and control signals are shown for the hip extensor (HE), hip flexor (HF), knee flexor (KF), and knee extensor (KE).

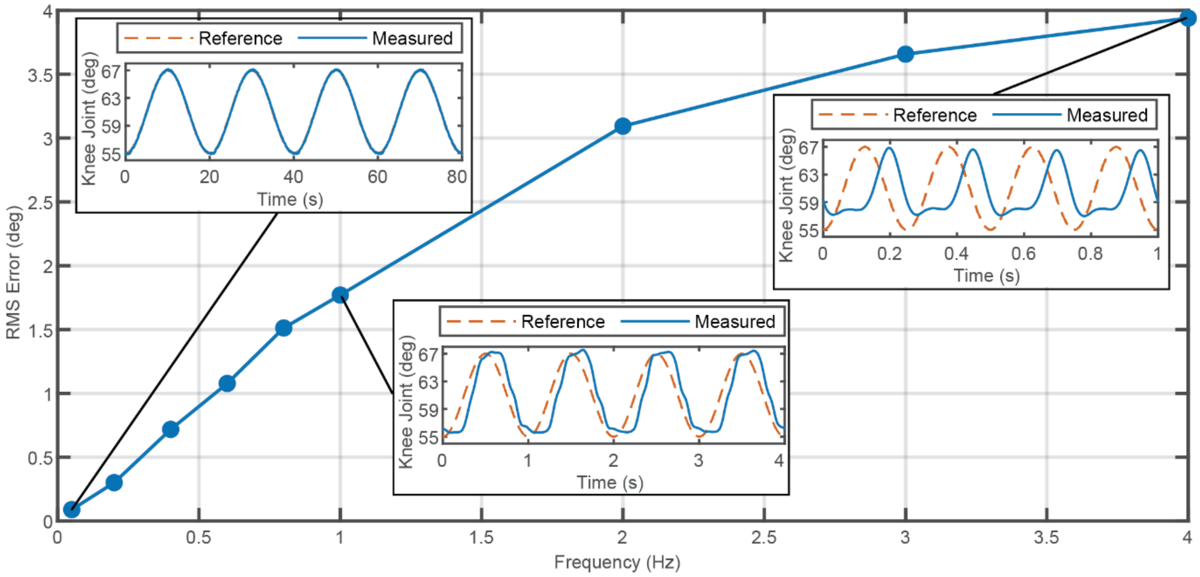


**Supplementary Figure 4 | Closed-loop control performance evaluation.** The leg's control performance in joint space is assessed using the root mean square (RMS) values of the measured joint angles as a function of the commanded joint position frequency (Equation 11). The joint angles are measured using a magnetic encoder in the joint. The result illustrates the leg's capability of tracking the specified joint positions at varied desired motion frequencies. Additionally, time evolution plots of the knee joint are included for three different frequencies: 0.05 Hz, 1 Hz, and 4 Hz. The results revealed that at lower frequencies, trajectory tracking is highly satisfactory. However, there was a noticeable increase in phase delay and deviation from the desired positions at higher frequencies.

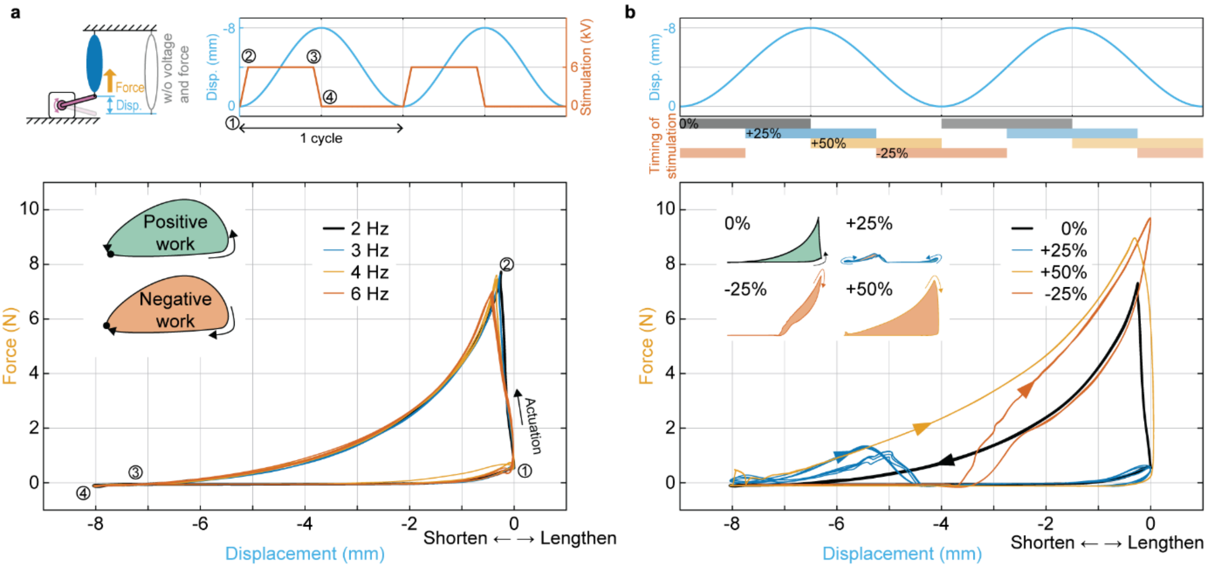


**Supplementary Figure 5 | Work loop analysis of the knee extensor muscle. a,** Experimental setup incorporates a muscle tester (310C-LR, Aurora Scientific) to evaluate work loops during vertical hopping at different frequencies. Five consecutive work loop cycles are shown for each frequency in steady-state conditions. The work loops show similar trajectories across different hopping frequencies. The muscle displacement during the hopping experiment was emulated by prescribing a specific sinusoidal displacement signal (8 mm peak-to-peak; equivalent to 5 % actuator strain) for this work loop analysis. The zero-point of the displacement was set as the initial length of the muscle without stimulation (0 kV). **b,** Impact of stimulation timing on work loop.

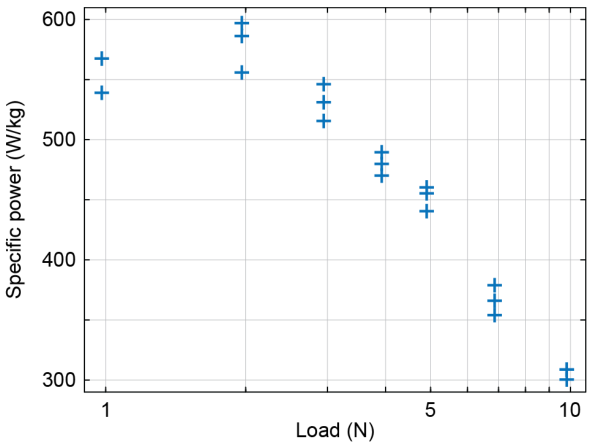


**Supplementary Figure 6 | Specific power of the Peano-HASEL artificial muscle.** We analyzed the peak specific power of our Peano-HASEL actuators using various weights (100 g, 200 g, 300 g, 400 g, 500 g, 700 g, 1000 g) following an analysis detailed in Kellaris et al. (40). The muscle recorded the highest specific power of 580 W/kg under around 2 N load.

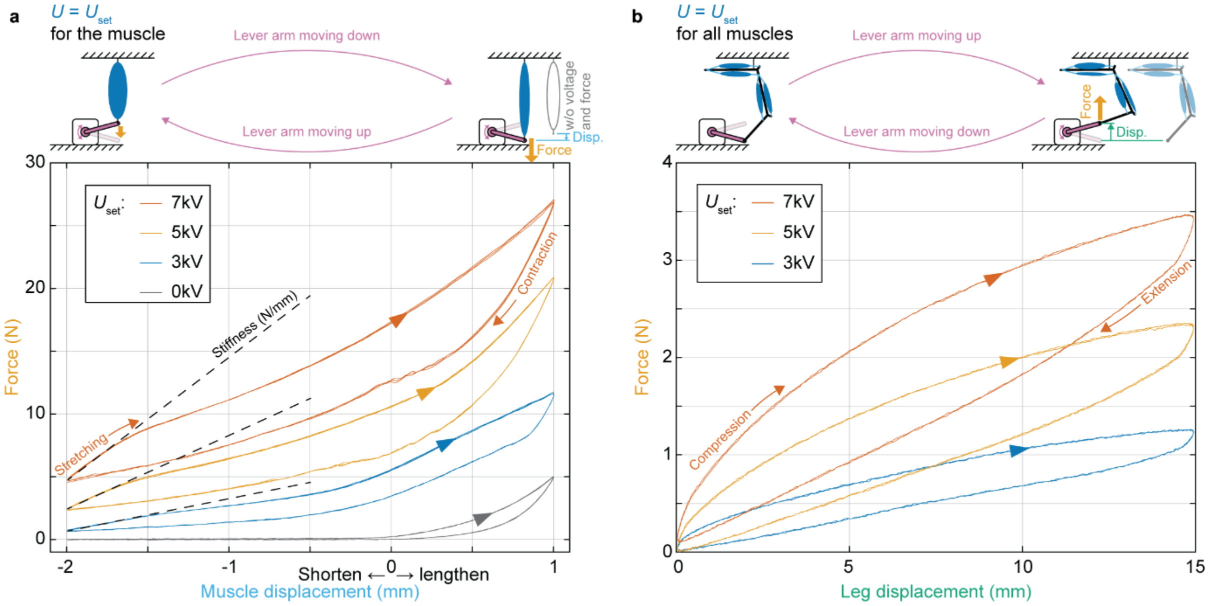


**Supplementary Figure 7 | Stiffness of the HASEL muscle and PELE changes proportionally to the applied voltage. a,** Experimental setup evaluates the stretching stiffness of the Peano-HASEL artificial muscle at different voltage levels using a muscle tester (310C-LR, Aurora Scientific). The slope angles of the trajectories represent the muscle stiffness. Higher voltage causes higher stiffness of the muscle. **b,** Experimental setup evaluates the compression stiffness of the leg using a muscle tester (310C-LR, Aurora Scientific). A higher applied voltage causes a higher leg stiffness.

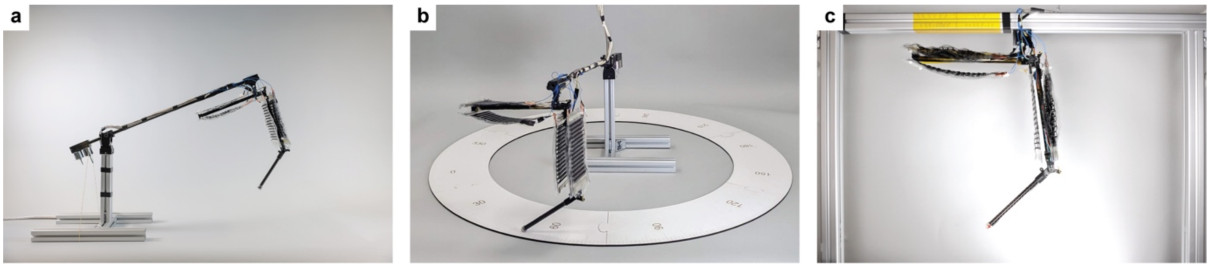


**Supplementary Figure 8 | Test setups used for our experiments. a,** A *1-DOF boom* was used for high jump tests. **b,** The *2-DOF boom* was used for all locomotion tests, allowing the leg to hop in a circular path. **c,** We used the *frame mount* for leg experiments without contact with the ground.

**Supplementary Table 1 | Motor – HASEL actuator equivalence.** While holding a constant position, a motor-driven system In HASEL actuators, power consumption while holding a posture does not relate to exerting torque.

| **Metrics** | **DC-motor** | **HASEL** |
|---|---|---|
| Output | Torque | Force |
| Control | Current | Voltage |
| Sensor | Series resistor | Parallel resistor |
| Power consumption during holding position | $P = VI = I^2R \propto \tau^2$ | $P = VI = \frac{Q}{C}\dot{Q} \sim 0$ |

**Supplementary Table 2 | Net cost of transport (COT) for varying levels of robot mass.**

| Leg mass (kg) | Weight Modification | Minimum net COT | Locomotion |
|---|---|---|---|
| 0.198 | Reduced | 0.69 | Hopping |
| 0.225 | Original | 1.79 | Hopping |
| 0.225 | Original | 0.73 | Crawling |
| 0.251 | Increased | 0.77 | Crawling |
| 0.329 | Increased | 0.82 | Crawling |
| 0.391 | Increased | 0.83 | Crawling |

**Supplementary Table 3 | Robots referenced in the cost of transport comparison in Fig. 5b.**

| # | Robot | Reference |
|---|---|---|
| 1 | HAMR-VP | (61) |
| 2 | HAMR-F | (62) |
| 3 | DASH | (63) |
| 4 | X2-VelociROACH | (64) |
| 5 | iSprawl | (65) |
| 6 | Series elastic leg (total) | (38) |
| 7 | Series elastic leg (net) | (38) |
| 8 | Slack damper leg | (66) |
| 9 | BirdBot | (39) |
| 10 | Oncilla | (67) |
| 11 | TITAN-XIII | (68) |
| 12 | Cornell Ranger | (69) |
| 13 | ARL Monopod II | (70) |
| 14 | Starl*ETH* | (71) |
| 15 | ANYmal | (3) |
| 16 | ETH Cargo | (72) |
| 17 | MIT Cheetah | (73) |
| 18 | MIT Cheetah 3 | (74) |
| 19 | ATRIAS 2.1 | (75) |
| 20 | BigDog | (76) |

**Supplementary Table 4 | Experiment setups.**

| Scope | # | Experiment | Setup |
|---|---|---|---|
| Controllability | 1 | Trajectory tracking | Frame mount |
| Dynamic locomotion | 2 | High jump | 1D-boom |
| Dynamic locomotion | 3 | Agile vertical hopping | 1D-boom |
| Dynamic locomotion | 4 | Rapid gait motion | Frame mount |
| Inherently adaptive locomotion | 5 | Hopping over varying terrains | 2D-boom |
| Inherently adaptive locomotion | 6 | Terrain-adaptive transition | 1D-boom |
| Inherently adaptive locomotion | 7 | Inherent soft landing | 1D-boom |
| Energy efficient locomotion | 8 | Energy efficient locomotion | 2D-boom |
| Energy efficient locomotion | 9 | Energy efficient squatting | 1D-boom |
| Self-sensed locomotion | 10 | Sensor-less obstacle detection | 2D-boom |

**Supplementary Table 5 | High voltage amplifiers used in the experiments.** The amplifiers are characterized based on their maximum voltage and current and their use in experiments numbered according to Supplementary Table 4.

| **Amplifier** | **Max. voltage (kV)** | **Max. current (mA)** | **Used experiments** |
|---|---|---|---|
| TREK 610E | 10 | 2 | 1, 3 to 10 |
| PolyK PK-HVA1005 | 10 | 5 | 1 to 5, 8, 10 |
| Matsusada AMPS-10B40 | 10 | 40 (DC), 120 (AC) | 2, 3, 6 |

**Supplementary Table 6 | Considered motion cycles in Fig. 3e.** The number of motion cycles indicates when a steady state has been reached.

| **Frequency (Hz)** | **Numbers of cycles** |
|---|---|
| 0.5 | 8 |
| 1 | 6 |
| 2 | 13 |
| 3 | 21 |
| 4 | 16 |
| 5 | 34 |
| 10 | 69 |

# References


61. Baisch, A. T. & Wood, R. J. Pop-up assembly of a quadrupedal ambulatory MicroRobot. *2013 IEEE/RSJ International Conference on Intelligent Robots and Systems* 1518–1524 (2013). doi:10.1109/IROS.2013.6696550

62. Goldberg, B. *et al.* Power and Control Autonomy for High-Speed Locomotion with an Insect-Scale Legged Robot. *IEEE Robotics and Automation Letters* **3,** 987–993 (2018).

63. Birkmeyer, P., Peterson, K. & Fearing, R. S. DASH: A dynamic 16g hexapedal robot. *2009 IEEE/RSJ International Conference on Intelligent Robots and Systems* 2683–2689 (2009). doi:10.1109/IROS.2009.5354561

64. Haldane, D. W. & Fearing, R. S. Running beyond the bio-inspired regime. *2015 IEEE International Conference on Robotics and Automation (ICRA)* 4539–4546 (2015). doi:10.1109/ICRA.2015.7139828

65. Kim, S., Clark, J. E. & Cutkosky, M. R. iSprawl: Design and Tuning for High-speed Autonomous Open-loop Running. *The International Journal of Robotics Research* **25,** 903– 912 (2006).

66. Mo, A., Izzi, F., Gönen, E. C., Haeufle, D. & Badri-Spröwitz, A. Slack-based tunable damping leads to a trade-off between robustness and efficiency in legged locomotion. *Sci Rep* **13,** 3290 (2023).

67. Spröwitz, A. T. *et al.* Oncilla Robot: A Versatile Open-Source Quadruped Research Robot with Compliant Pantograph Legs. *Frontiers in Robotics and AI* **5,** (2018).

68. Kitano, S., Hirose, S., Horigome, A. & Endo, G. TITAN-XIII: sprawling-type quadruped robot with ability of fast and energy-efficient walking. *ROBOMECH Journal* **3,** 8 (2016).

69. Bhounsule, P. A., Cortell, J. & Ruina, A. in *Adaptive Mobile Robotics* 441–448 (WORLD SCIENTIFIC, 2012). at <doi.org/10.1142/9789814415958_0057>

70. Ahmadi, M. & Buehler, M. Controlled passive dynamic running experiments with the ARL- monopod II. *IEEE Transactions on Robotics* **22,** 974–986 (2006).

71. Hutter, M., Gehring, C., Höpflinger, M. A., Blösch, M. & Siegwart, R. Toward Combining Speed, Efficiency, Versatility, and Robustness in an Autonomous Quadruped. *IEEE Transactions on Robotics* **30,** 1427–1440 (2014).

72. Günther, F., Shu, Y. & Iida, F. Parallel elastic actuation for efficient large payload locomotion. *2015 IEEE International Conference on Robotics and Automation (ICRA)* 823– 828 (2015). doi:10.1109/ICRA.2015.7139273

73. 73.Seok, S. *et al.* Design Principles for Energy-Efficient Legged Locomotion and Implementation on the MIT Cheetah Robot. *IEEE/ASME Transactions on Mechatronics* **20,** 1117–1129 (2015).

74. Bledt, G. *et al.* MIT Cheetah 3: Design and Control of a Robust, Dynamic Quadruped Robot. *2018 IEEE/RSJ International Conference on Intelligent Robots and Systems (IROS)* 2245– 2252 (2018). doi:10.1109/IROS.2018.8593885

75. Hubicki, C. *et al.* ATRIAS: Design and validation of a tether-free 3D-capable spring-mass bipedal robot. *The International Journal of Robotics Research* **35,** 1497–1521 (2016).

76. Raibert, M., Blankespoor, K., Nelson, G. & Playter, R. BigDog, the Rough-Terrain Quadruped Robot. *IFAC Proceedings Volumes* **41,** 10822–10825 (2008).
