# Supplementary material for: Electrohydraulic musculoskeletal robotic leg for agile, adaptive, yet energy-efficient locomotion
Source: Nat Commun. 2024 Sep 9;15:7634. doi: 10.1038/s41467-024-51568-3 (PMC11385520; doi:10.1038/s41467-024-51568-3)
Supplement: Supplementary file 3 — Description of Additional Supplementary Files [file 41467_2024_51568_MOESM3_ESM.pdf]

Description of Additional Supplementary Files

Supplementary Movie 1 A short preview of how the system operates and a summary of the results.

Supplementary Movie 2 Precise control experiments with the system.

Supplementary Movie 3 The system performing a high jump, agile vertical hopping, and rapid gait motion.

Supplementary Movie 4 Versatile hopping on varying terrain with solely open-loop control, terrain adaptive transition of locomotion, and inherently soft landing with tunable stiffness.

Supplementary Movie 5 Energy-efficient locomotion and squatting of the system. Supplementary Movie 6 Obstacle avoidance triggered by the selfsensing function of the muscle.
